# GOLF CARTS AS A TRANSPORTATION MODE: WHO USES THEM?

**DOI:** 10.1093/geroni/igad104.2178

**Published:** 2023-12-21

**Authors:** Anne Barrett, Hope Mimbs, Jessica Noblitt, Cherish Michael, Brianna Soulie

**Affiliations:** Florida State University, Tallahassee, Florida, United States; Florida State University, Tallahassee, Florida, United States; Florida State University, Tallahassee, Florida, United States; Florida State University, Tallahassee, Florida, United States; Florida State University, Tallahassee, Florida, United States

## Abstract

Golf carts are rising in popularity among older adults, not only for use on the course but also for traveling short distances. Alongside their rising popularity are increases in related injuries, which have been the focus of the relatively small literature on golf carts. Little is known, however, about the frequency or social patterning of older adults’ use of golf carts as a transportation mode. Using data from an online survey of Floridians aged 50 and older that was conducted between December 2020 and April 2021 (n=4,199), we used multivariate regression to examine five sets of predictors of golf cart use: sociodemographics, health, subjective aging experiences, transportation experiences, and social relationships. Sociodemographics predicting more frequent golf cart use included being younger, male, or white. More frequent golf cart use also was associated with lower levels of loneliness but was unrelated to physical health. Other predictors of more frequent golf cart use included reporting older age identities and more frequent walking. Also significant were social relationship measures. Results revealed more frequent golf cart use among married individuals and those interacting more frequently with friends – but less frequently with family members. By examining a variety of factors that may influence golf cart use, our study provides clues about how golf carts may relate to older adults’ transitioning from driving.

